# Key parameters for designing robust 2D and 3D spheroid models for *in vitro* atherosclerosis research

**DOI:** 10.1002/btm2.10736

**Published:** 2025-03-21

**Authors:** Ibukunoluwa Naiyeju, Stephanie Lehoux, Maryam Tabrizian

**Affiliations:** ^1^ Department of Biomedical Engineering McGill University Montreal Quebec Canada; ^2^ Lady Davis Institute for Medical Research Montreal Quebec Canada; ^3^ Faculty of Dental Medicine and Oral Health Sciences McGill University Montreal Quebec Canada

**Keywords:** Atherosclerosis, co‐culture model, *in vitro* models, model design, spheroid model

## Abstract

Atherosclerosis is a chronic, systemic, inflammatory disease associated with the build‐up of fatty deposits (“plaques”) in the arteries. A major global health burden, severe atherosclerosis progresses to ischemic heart disease, an underlying condition which can exacerbate the occurrence of fatal events such as heart attack and stroke. Over the past two decades, the use of *in vitro* models to study atherosclerotic phenomena has increased, with the goal of complementing clinical research for drug and therapy development. In particular, 2D co‐culture models, and in the last decade, 3D spheroid models have been developed to improve our understanding of the atherosclerotic disease mechanism. However, the existing literature lacks information on the relevant parameters which should be considered prior and during the design of these models to promote model robustness and enhance their biomimetic capacities. This review provides an overview of such key parameters, as well as future perspectives on how existing limitations in the field of cell‐based *in vitro* model design can be improved. It is expected that by carefully considering these parameters, researchers will be better equipped with the required knowledge to develop biomedically and clinically relevant *in vitro* models.

**Translational Impact Statement**

*In vitro* models are used in the field of tissue engineering to replicate the complex composition and functionality of native tissues and organs and study disease phenomena. Likewise, advancements to study atherosclerotic disease using cell‐based models (2D and 3D) have been made in the past two decades. In this review, we discuss these advancements and propose the key parameters to consider for the effective design of biomimetic atherosclerosis models. We also share future perspectives to promote the design of novel and reproducible cell‐based models.

## 1 INTRODUCTION

Atherosclerosis (AT) is a chronic, systemic, inflammatory disease associated with the build‐up of fatty deposits (“plaques”) in the arteries. An enormous global health burden, severe atherosclerosis progresses to ischemic heart disease, the leading cause of death in the world, and provokes ischemic strokes, the fifth cause of death in the world.^1^ Corroborating this, the World Health Organization (WHO) has reported that cardiovascular diseases (CVDs) precipitated by atherosclerosis are now the leading cause of deaths globally.^2^ Endothelial dysfunction has been reported to catalyze the onset of atherosclerosis, as a dysfunctional endothelium is leaky and permits the entry and accumulation of low‐density lipoproteins (LDL) and cholesterol in the subendothelial space.^3^ The presence of these foreign macromolecules in the subendothelial space initiates an inflammatory signaling pathway, which leads to the expression of adhesion molecules (vascular cell adhesion molecule 1 (VCAM‐1), intercellular adhesion molecule 1 (ICAM‐1), P‐Selectin, and E‐Selectin) to which circulating monocytes bind.^3^, ^4^ Upon gaining access into the sub‐endothelial space, these monocytes differentiate into macrophages, take up the lipids, and transform into foam cells. As the inflammatory process continues, more immune cells are recruited, ultimately leading to an increase in plaque size.

**FIGURE 1:**
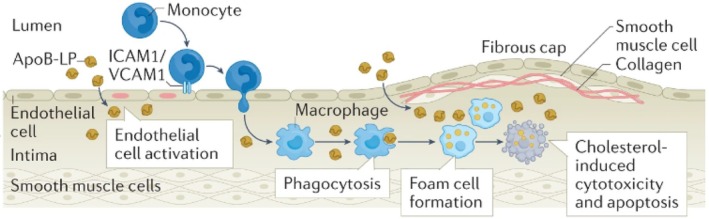
Schematic to show the initiation of atherosclerosis. Reproduced from Reference 5.

Both macrophages and vascular smooth muscle cells (SMCs) within the lesion accumulate lipids, transforming into foam cells. SMCs can also synthesize collagen, which is used to form the fibrous cap that shields the plaque from rupture. A portion of cells within the plaque undergo apoptosis, and failure to uptake such cells and cell debris through defective efferocytosis results in the development of a necrotic lipid core. As the plaque grows, the amount of oxygen supplied to the intima from the lumen becomes limited, causing local hypoxia. To compensate for this deficit, angiogenesis is triggered in the vasa vasorum (the microvascular network which supplies nutrients to arterial walls), leading to the formation of new microvessels, known as “neovessels.”^4^ These neovessels are prone to leakage and have been reported to facilitate the recruitment of additional immune cells to the plaque.^4^ A notably lethal group of plaques—“vulnerable” atherosclerotic plaques—are characterized by active inflammation, large lipid cores, and thin fibrous caps prone to rupture.^4^ This instability within the atherosclerotic lesion is sustained by the pro‐inflammatory state (Figure 1).^4^, ^5^

**FIGURE 2:**
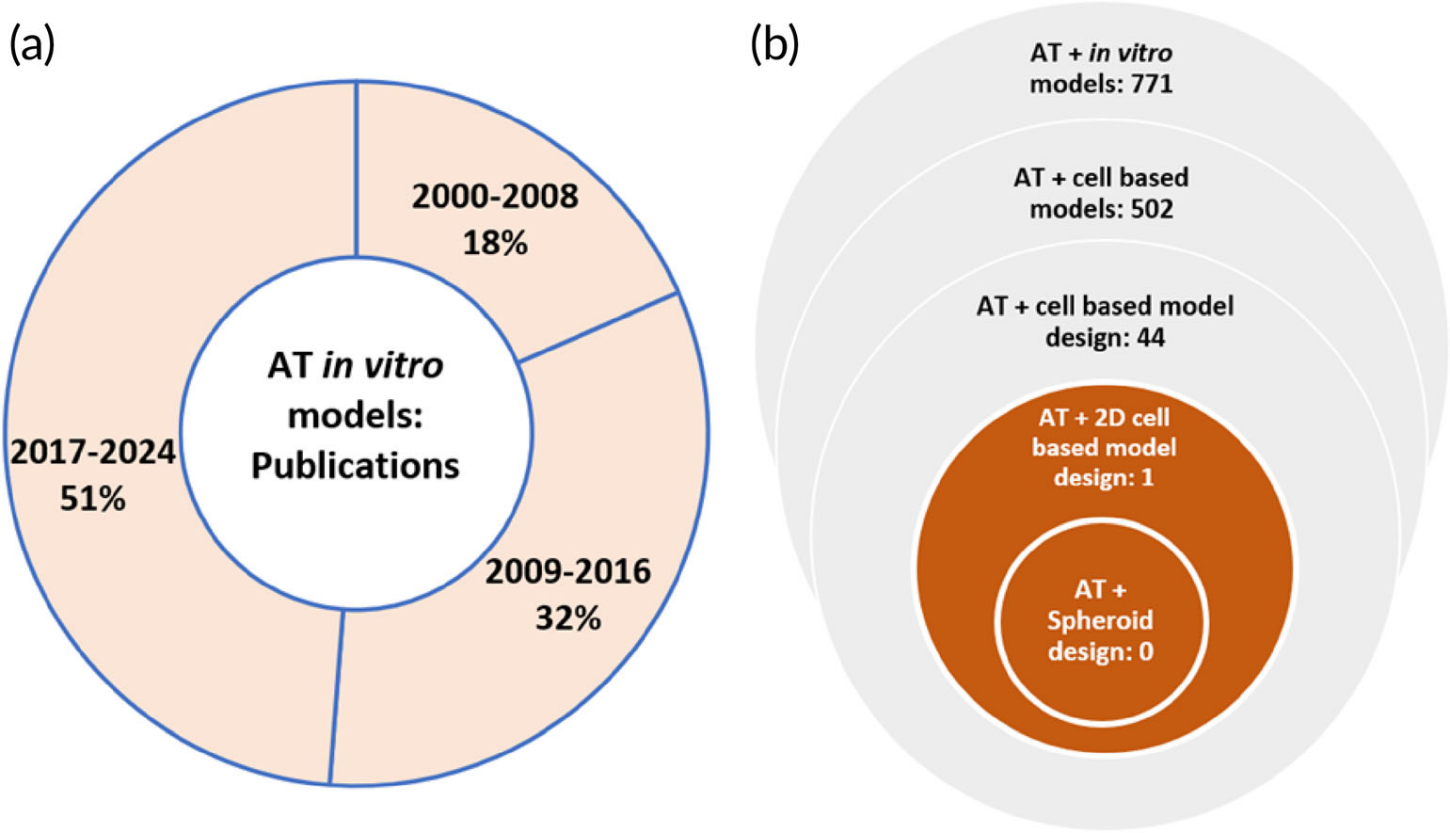
(a) Donut plot to show the rising number of original research articles in the field of atherosclerosis (AT) *in vitro* models. A search was performed on SCOPUS using the keywords “Atherosclerosis” and “*in vitro* models.” Results were limited to research articles written in the English language from 2000 to 2024. A total of 5,506 articles were found. (b) Schematic to show the novelty of this review. A SCOPUS search was performed using the keywords shown in the schematic. Niche areas focused on in this review article are highlighted at the core.

Thus, atherosclerosis research has shifted towards the adoption of a “predictive” approach, where vulnerable plaques can be identified before the occurrence of adverse cardiovascular events.^4^ Moreso, advancements have been made in the field to replicate the environment of the atherosclerotic plaque using cell‐based *in vitro* models. These models represent a reduction in the time and cost associated with the use of in vivo models and offer other advantages such as reproducibility and applicability for high‐throughput screening.^6^ The use of *in vitro* models in the field of tissue engineering is now a common practice to replicate the complex composition and functionality of native tissues and organs, study disease phenomena and screen potential drug molecules.^7^, ^8^ *In vitro* models have been developed to study the physiology of the blood–brain barrier and peripheral nerve regeneration^9^, ^10^ and the pathophysiology of other disease conditions such as traumatic brain injury, tuberculosis, diabetic neuropathy, kidney disease, and various cancers.^9^, ^10^, ^11^, ^12^, ^13^, ^14^, ^15^, ^16^, ^17^ Similarly, cardiac tissues and blood vessels have been fabricated to mimic their native equivalents by three‐dimensional (3D) printing techniques, as research advancements emerge in the development of biocompatible 3D scaffolds, vascular models, and microchannels for tissue engineering applications.^18^, ^19^, ^20^, ^21^, ^22^, ^23^ The adoption of *in vitro* models to better understand the underlying mechanism of atherosclerosis is more recent, and the number of publications reporting on the original research in this field keeps increasing (Figure 2).

**FIGURE 3:**
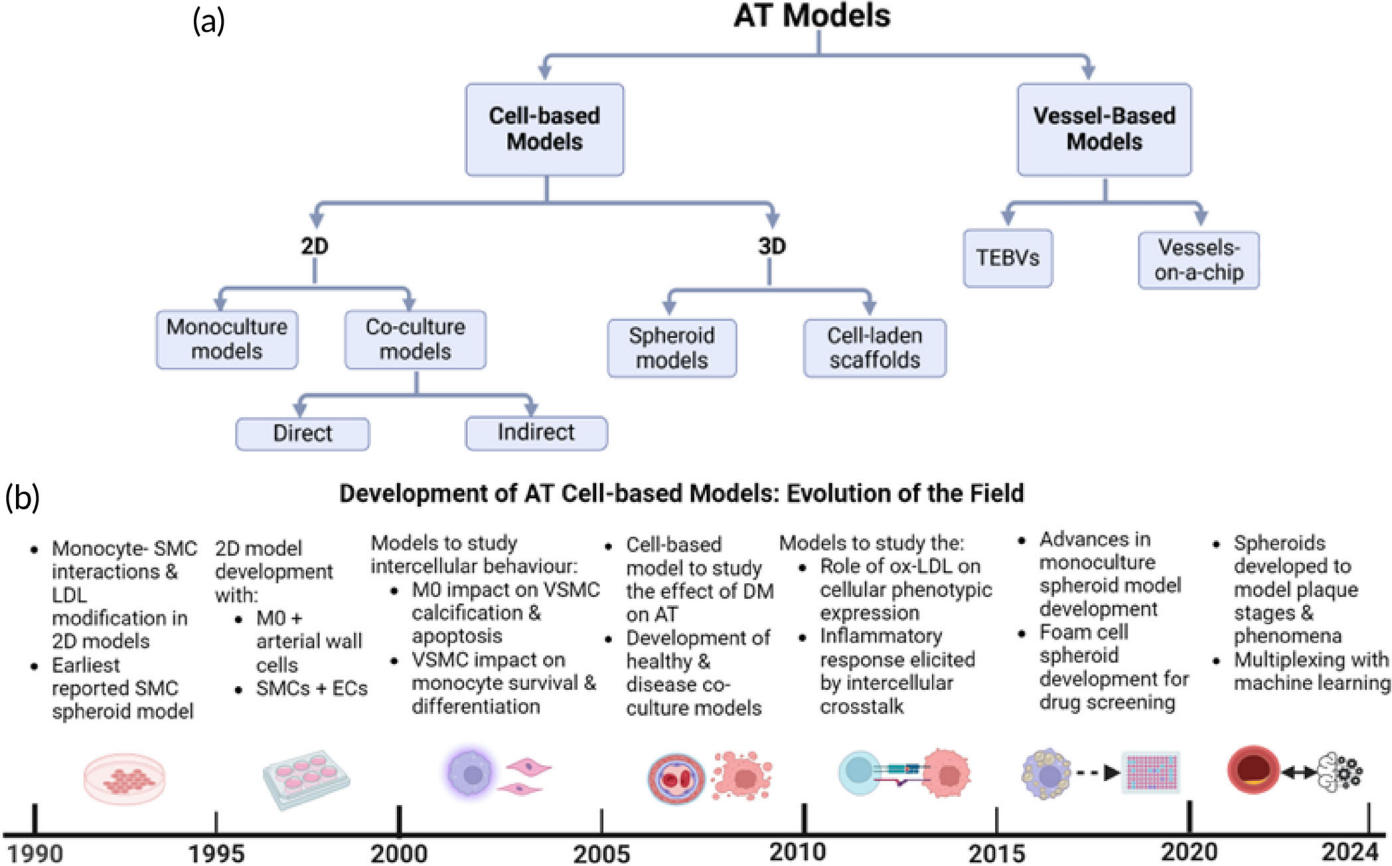
(a) Categories of Atherosclerosis (AT) models. (b) Evolution of AT cell‐based models for the study of atherosclerotic phenomena.

Cell‐based models of atherosclerosis may be classified into two broad categories according to their complexity: two‐dimensional (2D) or three‐dimensional (3D). 2D atherosclerosis models include 2D culture models (monoculture or co‐culture model) while 3D models include spheroids and 3D cell‐laden scaffolds. Vessel‐based models include vessels‐on‐a‐chip and tissue engineered blood vessels (TEBVs).^24^, ^25^, ^26^ To date, the shift towards the adoption of 3D models has been informed by a drive to overcome the limitations of traditional 2D cell‐based models in replicating the complexity and heterogeneity of the native in vivo tissue. Thus, 3D models are beneficial to achieve higher biomimicry of cell–cell and cell‐ECM interactions, gene expression, cellular heterogeneity, and microarchitecture of the native tissue.^24^, ^27^, ^28^, ^29^ Review articles are available about 3D cardiac cell‐based models, cell‐laden hydrogel constructs, TEBVS, and vessels‐on‐a‐chip.^6^, ^30^, ^31^, ^32^, ^33^ However, a review which discusses the parameters which impact the robust design of atherosclerosis cell‐based models is lacking (Figure 2). To address this knowledge gap, we present some fundamental insights about the evolution in the field (Figure 3) and discuss key findings from existing 2D cell co‐culture and 3D spheroid models of atherosclerosis. Furthermore, we examine the key parameters to consider when designing biomimetic cell‐based models of the atherosclerotic plaque and provide some directions for future research.

## 2 2D CELL‐BASED MODELS FOR ATHEROSCLEROSIS RESEARCH

Three main approaches can be used to establish co‐culture models in 2D: direct cell culture, indirect cell culture, and the use of gel‐based scaffolds.^34^, ^35^ In direct models, intercellular interactions and crosstalk are facilitated due to direct contact of the cells with one another. In indirect models, the cells are separated by a transwell insert or by a collagen or fibrin scaffold.^35^ In either case, cells can be either target cells, which are directly involved in the pathological process being studied, or assisting cells which aid or influence the action of the target cells.^31^ Existing 2D cell‐based models have been mostly developed by the co‐culture of two or more cell types to study sub‐endothelial transmigration, inflammatory response and its effect on SMC functionality, and other intercellular interactions, including the effect of diabetic metabolic products on SMC‐monocyte adhesion. These models are discussed in the following sections.

### 2.1 2D cell models studying sub‐endothelial transmigration

A summary of key co‐culture models developed to study subendothelial transmigration is provided in Table 1.

**TABLE 1:** Co‐culture models developed to study sub‐endothelial transmigration.

Cells	Co‐culture approach	Duration	Study focus	Ref.
HASMCs HUVECs Freshly isolated neutrophils from healthy donor	Direct HUVECs seeded on the surface of fibrin gel, followed by incubation with human LDL & neutrophils.	Up to 72 h	Endothelial adhesion & neutrophil transmigration within a neo‐intima model.	36
HUVECs Donor‐obtained PBMCs	Direct PBMCs added to confluent HUVEC monolayers grown on LDL‐containing collagen matrices. Co‐culture subsequently stimulated with TNF‐α.	Up to 10 days	Trans‐endothelial migration of PBMCs into collagen matrices.	37
HASMCs HAECs Human PBMs	Indirect SMCs seeded into a 24‐well plate. At day 2, the confluent monolayer was treated with fibronectin before seeding with ECs. Co‐cultures were supplemented with LDL.	24–48 h	Effect of LDL incubation & leumedin on monocyte migration into the subendothelial space.	38
Rabbit aortic ECs Rabbit aortic SMCs Human peripheral blood monocytes	Direct SMCs cultured on a chemotaxicell filter for 10–14 days to facilitate ECM secretion. Ox‐LDL added to the ECM, ECs & monocytes seeded on top of ECs.	4–7 days	EC layer functionality, monocyte transmigration & SMC contractility in the co‐culture model.	39
HASMCs HAECs Human blood monocytes	Direct SMCs seeded on top of a gelatinized polycarbonate membrane; ECs seeded on top of them.	24–48 h	Monocyte transmigration & endothelial adhesion in the presence of LDL.	40
HASMCs HAECs Human blood monocytes	Indirect SMCs seeded onto the gelatinized surfaces of polycarbonate filters. At day 2, the SMCs were covered with a fibronectin‐collagen layer; HUVECs seeded on top of the collagen layer.	24–48 h	LDL transport and monocyte migration into the sub‐endothelial space.	41
HUASMCs HUVECs Mono‐Mac‐6 monocytes	Indirect SMCs cultured in a fibrin solution to create a baseline fibrin scaffold. After 2 weeks, HUVECs were seeded on top of the scaffold.	4–6 weeks	ECM secretion, lipid deposition & monocyte infiltration into the sub‐endothelial space.	42

As shown, both direct and indirect approaches were used to coculture endothelial cells (EC) predominantly with SMCs and study lipid or monocyte migration into the sub‐endothelial space at various time points. For instance, using a human neo‐intima SMC‐EC model, neutrophil infiltration into the sub‐endothelial layer was observed for up to 72 h, reportedly mediated by SMC‐secreted IL‐8.^36^ Furthermore, the observed neutrophil infiltration enhanced the release of matrix degrading enzymes, namely matrix metalloproteinase (MMP)‐8 and elastase, within the model and increased EC apoptosis. A similar cell migration pattern was observed with a co‐culture model where monocytes traversed the endothelial layer into the underlying collagen matrix system, became differentiated to macrophages, and expressed a foam cell‐like morphology post‐exposure to ox‐LDL & TNF‐α.^37^

### 2.2 2D cell models investigating inflammatory response

Cell‐based models have also been utilized to study the expression of inflammatory cytokines and matrix metalloproteinases. Inflammation plays a frontline role in the progression of atherosclerotic disease,^43^ and different models have been developed to investigate the nature of inflammatory responses provoked by the interaction of immune cells (either donor‐obtained or commercially available) with SMCs. The highlights of these studies are provided in Table 2.

**TABLE 2:** Co‐culture models developed to study inflammatory response.

Cells	Co‐culture approach	Duration	Study focus	Ref.
HVSMCs Monocytes & lymphocytes isolated from blood	Direct Co‐culture of mononuclear cells (+/−LPS) with SMCs; ratio 1:1.	24 h	IL‐6 & MCP‐1 expression in a direct co‐culture model.	44
HASMCs Blood‐isolated monocytes	Direct LPS‐activated monocytes added to confluent SMCs monolayers.	18 h	Intracellular ROS production & protein expression (resistin, SOCS, STAT3, actin).	45
HASMCs U937 monocytes	Direct Monocytes (LPS‐activated & non‐LPS activated) added to confluent SMC monolayers.	18 h	Effect of CX3CL1‐CX3CR1 binding on TNF‐α, IL‐1β, IL‐6, MMP‐2, & MMP‐9 expression.	46
Peripheral blood obtained human monocytes Human SMCs	Direct SMCs & monocytes co‐culture. Indirect SMCs exposed to monocyte‐conditioned media or monocytes treated with SMC‐conditioned media.	3 days	Role of soluble mediators in MMP production.	47
HASMCs THP‐1 monocytes	Direct THP‐1 treated with PMA seeded on confluent SMC monolayers. Indirect THP‐1 cultured on the upper chamber of a cell culture insert; SMCs in the lower chamber.	Up to 48 h	Collagenolytic activity & expression of MMP‐1, TIMP‐1, IL‐1β, IL‐6 and TNF‐α within the model.	48
THP‐1‐derived macrophages HCASMCs HCAECs	Indirect SMCs cultured on the basal side and ECs in the apical side; insert transferred to wells containing of a LPS‐pre‐treated THP‐1.	20 h	Role of SMCs in promoting macrophage‐EC inflammatory crosstalk.	51
THP‐1‐derived macrophages HCASMCs HCAECs	Indirect SMCs cultured on the basal side of a transwell insert; ECs cultured in the apical side. Inserts transferred to wells containing LPS‐pre‐treated THP‐1 macrophages.	Up to 24 h	Interaction between vascular & immune cells within a triple cell co‐culture model.	50
HASMCs THP‐1 derived macrophages	Indirect SMCs cultured in the lower chamber of a Transwell insert, THP‐1 macrophages on the upper chamber.	Up to 24 h	Role of ficolin‐2 in macrophage‐SMC crosstalk.	49

Soluble mediators such as IL‐1, IL‐6 and TNF‐α have been shown to synergistically aid the expression of pro‐inflammatory cytokines IL‐6 and MCP‐1 *in vitro*.^44^ The influence of reactive oxygen species (ROS) and resistin, key contributors to the progression of inflammation in cardiovascular diseases, has also been studied. Resistin increases SMC & EC expression of pro‐inflammatory mediators, while ROS are signaling molecules which mediate cell differentiation and apoptosis.^45^ Increased SMC ROS production and monocyte resistin expression was observed in a model of SMC co‐culture with LPS‐activated monocytes due to activation of the STAT3 transcription factor.^45^ Similarly, in a direct monocyte‐SMC co‐culture model, chemokine fractalkine binding to its receptor (CX3CL1/CX3CR1) was enhanced, and an increase in IL‐1β, IL‐6, TNF‐α, MMP‐2 and MMP‐9 expression was observed.^46^

The relevance of paracrine signaling for metalloproteinase synthesis has been investigated in a monocyte‐SMC model, with MMP secretion being elevated when the SMCs were treated with monocyte‐conditioned media.^47^ In contrast, when the monocytes were treated with SMC‐conditioned media, no observable increase in MMP production was noticed, a finding attributed to the absence of monocyte‐secreted IL‐1 in the SMC‐conditioned media.^47^ MMP‐1 production and collagenolytic activity were also significantly enhanced over time in a co‐culture model of SMCs and PMA‐stimulated THP‐1 monocytes.^48^ Interestingly, the peak MMP‐1 production was observed at a THP‐1 monocyte: SMC co‐culture ratio of 1:1 and attributed to being due to the co‐culture of both cells.^48^

**FIGURE 4:**
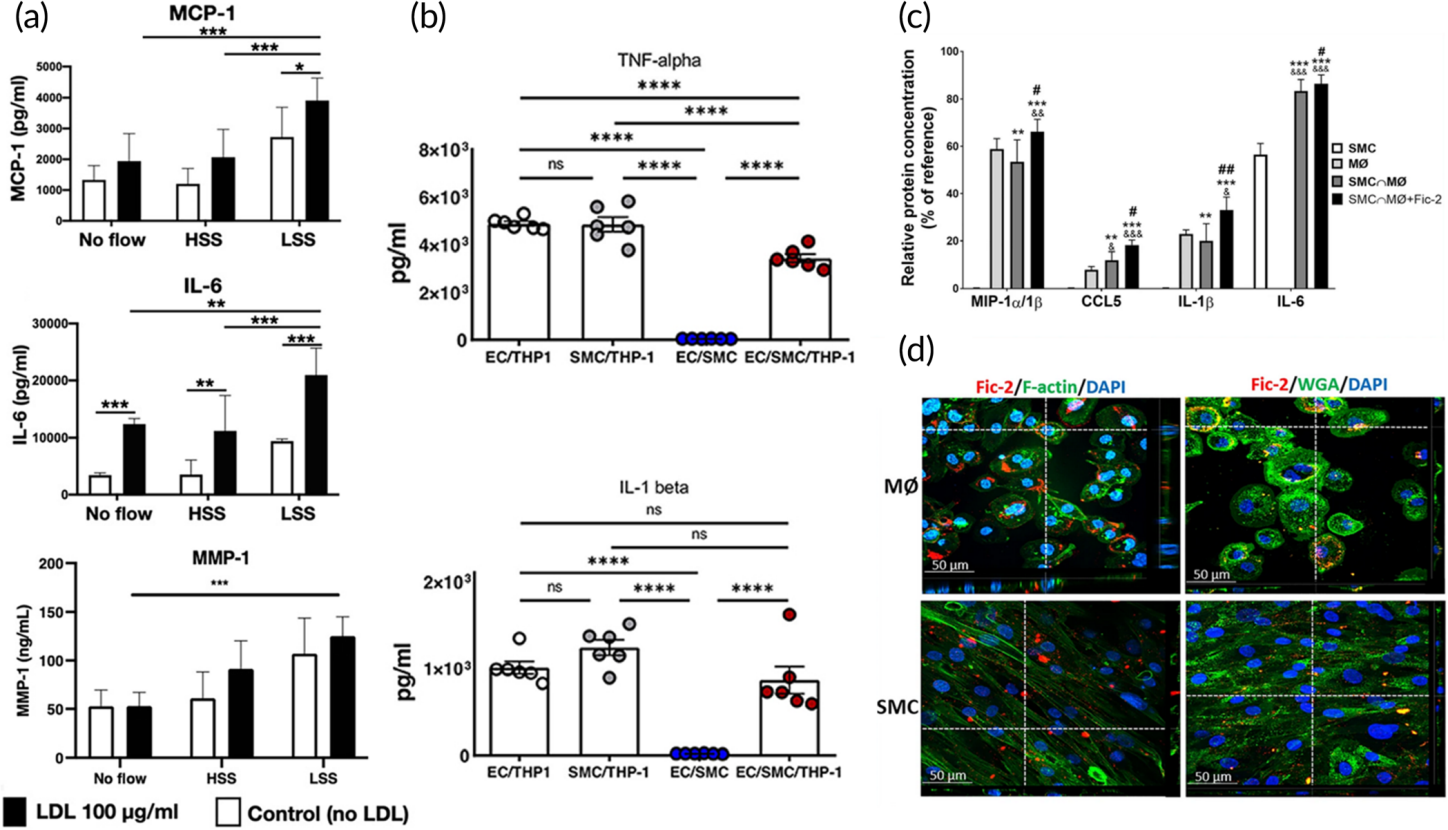
Expression of pro‐inflammatory mediators in 2D co‐culture models of atherosclerosis. (a) Increased expression of MCP‐1, IL‐6and MMP‐1 in a THP‐1‐SMC_EC co‐culture model exposed to low shear stress (5 ± 3 dynes/cm^2^) and LDL. Controls were exposed to high shear stress (30 ± 3 dynes/cm^2^) or no flow (static control). *p < 0.05; **p < 0.01; ***p < 0.001. Reproduced from Reference 52. (b) Expression of pro‐inflammatory cytokines (TNF‐α & IL‐1β) in co‐culture & triple‐cell models of atherosclerosis after 24 h. *p <0.05, **p <0.01, ***p <0.001, ****p <0.0001. *n* = 6 co‐cultures. Reproduced from Reference 50. (c) Dot‐blot quantification of MIP‐1α/1β, CCL5, IL‐1β and IL‐6 protein expression when the SMC‐macrophage (MØ) co‐culture model is exposed to ficolin‐2. *p <0.05, **p <0.01, ***p <0.001 vs. SMC; &*p* <0.05, &&*p* <0.01, &&&*p* <0.001 vs. MØ and # *p* <0.05, ^##^ *p* <0.01 vs. SMC⋂MØ (*n* = 3). Reproduced from Reference 49. (d) Confocal microscopy image showing the expression of ficolin‐2 post‐treatment of macrophages (MØ) and SMCs with the recombinant protein for 24 h. Stains: Phalloidin‐FITC to label F‐actin; AlexaFluor488‐wheat germ agglutinin (WGA) to label cell membrane; DAPI to label nuclei. Reproduced from Reference 49.

A study conducted to elucidate the role of ficolin‐2 (a protein detected in the human carotid atherosclerotic plaque) in macrophage‐SMC crosstalk revealed an increased expression of the pro‐inflammatory cytokines (CCL5, IL‐1β, IL‐6, and macrophage inflammatory protein (MIP)‐1β) (Figure 4).^49^ Likewise, similar elevation of IL‐1β and TNF‐α expression was observed also in a co‐culture model (of SMCs & THP‐1 macrophages) and triple‐cell model (of SMCs, ECs & THP‐1 macrophages) (Figure 4).^50^ These triple‐cell (3‐cell) 2D models have been developed more recently to study the impact of cellular crosstalk on the release of key inflammatory mediators.^50^, ^51^ For example, production of the JAK3 enzyme, a downstream kinase which is activated by pro‐inflammatory cytokines, was notably induced in a 3‐cell model fabricated with LPS‐stimulated THP‐1 macrophages, SMCs and ECs, compared with the 2‐cell model fabricated with LPS‐stimulated THP‐1 macrophages and ECs.^51^

Likewise, inflammatory gene expression (including genes involved in JAK/STAT, Jun and NFκB signaling) and cytokine production (CXCL1/GROα and IL‐6) were observed in the 3‐cell model.^51^ These effects were attributed to the influence of SMCs on the other cell types within the 3‐cell model, and thus, the authors advocated the development of cell‐based models with triple cell types (macrophages, SMCs, and ECs) to obtain a more accurate understanding of the immune cell–vascular cell crosstalk at the molecular level.

### 2.3 2D cell models assessing the influence of inflammatory cells on SMC functionality

Co‐culture models investigating the influence of inflammatory cells (such as monocytes, macrophages and lymphocytes) on SMC functionality parameters including proliferation, calcification, and apoptosis at different time points have also been developed. Studies show that mediators produced by inflammatory cells can affect SMC functionality (Table 3).

**TABLE 3:** Co‐culture models developed to study SMC proliferation, calcification and apoptosis.

Cells	Co‐culture approach	Duration	Study focus	Reference
THP‐1 monocytes HVSMCs	Direct HVSMCs & THP‐1s co‐cultured in IFN‐γ & 1α,25‐dihydroxyvitamin D_3_ presence	4 days	Role of oncostatin M & TNF‐α in ALP upregulation in SMCs.	53
Human SMCs Mouse THP‐1 P388D1	Indirect Co‐cultures performed in 6‐well transwells.	10 days	Vitamin D receptor activator effect on SMC calcification.	54
Monocytes PBMCs HASMCs HCASMCs T‐lymphocytes	Direct Monocytes/macrophages & SMCs cocultured together.	8 days	Role of Fas‐ligand/Fas interaction.	55
Human monocytes SMCs	Direct Monocytes and VSMCs (+/− m‐CSF).	48 h	Role of Mac‐1 & ICAM interaction.	56
Commercial monocytes Donor heart SMCs	Direct Freshly isolated monocytes and SMCs, ratio 3:1, respectively (+/−m‐CSF).	72 h	Role of cell contact & m‐CSF activation of monocytes.	57
Freshly isolated human peripheral blood monocytes. HASMCs	Indirect Filter‐separated co‐culture: Monocytes cultured on top of filter inserts transferred to SMC‐containing wells. Dialysis membrane‐separated co‐culture: Monocytes cultured on top of filter inserts, a 12 kDa cut‐off dialysis membrane was placed around the inserts and transferred to wells containing SMCs.	40 h to 6 days	Influence of cyclooxygenase products & prostaglandins on SMC proliferation & growth.	58
Bovine aortic ECs Bovine aortic SMCs	Indirect *Bilayer model*: Cell culture insert containing ECs and SMCs seeded on opposite sides into each well of a 6‐well plate. *Conditioned media model*: ECs seeded at the bottom of the well; SMCs seeded on top of the cell culture insert.	Up to 14 days	Changes in SMC cellular morphology & proliferation in either model.	59
C57BL/6 and ApoE KO mice bone marrow derived macrophages C57BL/6‐derived aortic SMCs from	Indirect Macrophages (M1 or M2)‐conditioned medium added to SMCs in culture wells.	48 h	Effect of conditioned media on VSMC proliferation.	60
HCSMCs Peripheral blood‐isolated monocytes	Direct Monocytes co‐culture with the VSMC monolayer, ratio 3:1.	24–36 h	Effect of urokinase‐type plasminogen activator (uPA)/uPA receptor on VSMC growth inhibition.	61
Human peripheral blood macrophages HASMCs HCmSMCs HCarSMCs HCMED1‐E6 VSMCs	Direct Macrophages added to SMC monolayers; co‐culture ratio of macrophages: VSMC 4:1 or 8:1.	8 days	Role of nitric oxide & TNF‐α in VSMC apoptosis induction & role of anti‐TNF‐R1/R2 receptors in VSMC apoptosis inhibition.	62

In a co‐culture model of human peripheral blood monocytes and aortic SMCs, the effect of monocyte/macrophage‐released factors on SMC proliferation was studied.^58^ In this indirect model, monocytes were first cultured on top of filter inserts containing 12 kDa cut‐off dialysis membranes before their transfer to SMC‐seeded wells. Results showed that SMC proliferation was inhibited by membrane‐permeable growth‐inhibitory factors produced by the monocytes. This inhibitory effect was enhanced when the monocytes produced cyclooxygenase products including prostaglandins (PGE_1_ and PGE_2_) but reduced when the monocytes matured to macrophages. A similar model design (incorporating the 12 kDa membrane) was adopted by Fitzsimmons et al. to elucidate the impact of monocyte co‐culture on SMC procollagen production.^63^ The authors found that procollagen amounts in the culture medium were reduced by monocyte‐produced prostaglandins. However, when a cyclooxygenase inhibitor (indomethacin) was introduced, it competitively inhibited the prostaglandins and promoted a higher level of procollagen secretion.

Co‐culture of SMCs with macrophages can also elicit an effect on SMC calcification. Macrophages stimulated with 1α,25‐dihydroxyvitamin D_3_ (a vitamin D metabolite) produce oncostatin M when co‐cultured with SMCs, an agent which induces a higher expression of alkaline phosphatase (ALP) in SMCs & promotes their calcification.^53^ Vitamin D receptor activators—specifically calcitriol and paricalcitol—can also induce a similar calcification in SMCs.^54^ SMC apoptosis, however, is mediated by Fas/Fas‐ligand interactions on the surface of inflammatory cells (monocytes, macrophages & T‐lymphocytes).^55^ Thus, at low co‐culture ratios (monocyte: SMC 1:1 to 2:1), up to 50% cytotoxicity to the SMCs is achieved, and as the monocyte content of the co‐culture system is increased, higher SMC apoptosis occurs. Likewise, m‐CSF can increase SMC apoptotic index via Mac‐1 receptor activation.^57^ As m‐CSF‐activated monocytes express the Mac‐1 receptor which binds to SMC‐expressed intracellular adhesion molecule‐1 (ICAM‐1), the Fas–FasL pathway is activated, causing SMC apoptosis.^56^

### 2.4 2D cell models to study intercellular interactions & the effects of diabetic metabolic products

2D cell‐based models have further been developed to study intercellular interactions such as monocyte‐SMC binding, platelet adhesion, cellular phenotypic expression under different culture conditions (different media, presence or absence of LDL, shear stress), and the effect of diabetic metabolic products on SMC‐monocyte adhesion and differentiation. As shown in Table 4, most of these models have been direct models, which allow the cells to interact closely with one another similar to how they behave in their native microenvironment. Some of these studies show the prospects of cell‐based models for preclinical investigations into the effect of diabetes, a comorbidity which can complicate atherosclerosis. For example, the role of high glucose (HG) and advanced glycation end products (AGEs) on SMC‐monocyte adhesion and monocyte differentiation was investigated by Meng et al.^64^ The hyperglycaemic conditions induced with a high dose of glucose (25 mM for 7 days) enhanced monocyte‐VSMC binding, with key roles played by the chemokines MCP‐1 and CX_3_CL1 to enhance intercellular binding.^64^ Similarly, the effect of AGEs on SMC proliferation was investigated in another direct co‐culture model of SMCs, HUVECs and THP‐1 monocytes co‐cultured in the presence of 100 μg/ml of glycol‐AGEs for 24 h.^65^ The accumulation of AGEs in blood vessels contributes to the complication of diabetic disease, and unsurprisingly, the presence of AGEs in this co‐culture system was associated with a rise in pro‐inflammatory IL‐6 and MCP‐1 cytokine expression. A similar rise in the expression of pro‐inflammatory cytokines (IL‐6 and MCP‐1) as well as the matrix metalloproteinase MMP‐1 has been observed as a result of exposing a triple‐cell co‐culture model of ECs, SMCs and THP‐1 macrophages to low shear stress (Figure 4).^52^ Taken together, these studies show the prospects of cell‐based models for elucidating the molecular mechanisms of atherosclerosis progression.

**TABLE 4:** Co‐culture models to study intercellular interactions and the effect of diabetic metabolic products.

Cells	Co‐culture approach	Duration	Study focus	Ref.
HVSMCs THP‐1 monocytes PBMCs	Direct Monocytes added to confluent or fixed HVSMC monolayers.	Up to 6 h	Role of co‐culture with SMCs on monocyte survival.	66
HASMCs Human coronary SMCs HAECs	Direct SMCs cultured in SMC growth medium, then switched to differentiation medium for 36–48 h. ECs seeded on SMCs and medium changed to EC‐SMC co‐culture medium after 24–36 h.	Over 30 days	Phenotypic EC & SMC expression in different culture media treated with differentiation medium supplements (TGF‐β1 & heparin).	67
HUVECs Human SMCs Human platelets	Indirect ECs and SMCs cultured on opposite sides of porous inserts.	48 h	Effect of TNF & TGF‐β_1_ on platelet adhesion to ECs co‐cultured with SMC.	68
Donor‐obtained ECs Donor‐obtained SMCs	Direct ECs cultured on top of SMCs at a co‐culture ratio of ~3:1 (EC:SMC).	72 h	Effect of direct SMC‐EC co‐culture on angiogenic factor expression.	69
Donor peripheral blood mononuclear cells HVSMCs HAECs	Direct Fluorescence‐labeled‐acetylated LDL‐loaded macrophages added to SMCs; co‐culture ratio 3:1.	Up to 28 days	LDL transfer from macrophages to VSMCs & phenotypic switch of SMCs.	70
Bovine aortic SMCs & ECs Mouse P388D1 macrophages Human monocytes derived from heparinized blood	Direct Human monocyte‐derived macrophages seeded on SMCs. *SMC+ P388D1 macrophages*: Macrophages seeded on top of confluent SMCs. *SMC* + *EC*: Confluent SMCs treated with 0.1% fibronectin for 5 min before SMCs were added.	SMC: 5 days SMC: P388D1: 24 h SMC:EC: 4 days	Cholesteryl ester (CE) synthesis in co‐culture systems.	71
HASMCs HAECs Human‐blood obtained monocytes	Indirect SMCs seeded onto the bottom of the wells of a 96‐well plate and cultured for 3 days. ECs seeded on top of the matrix post‐ECM production.	48 h	Heme oxygenase‐1 (HO‐1) effect on monocyte chemotaxis in LDL‐containing co‐cultures.	72
HVSMCs THP‐1 monocytes	Direct Monocytes added to wells containing confluent monolayers of serum‐depleted SMCs.	30 min	Protein & chemokine expression post‐monocyte‐SMC binding.	64
HASMCs HUVECs THP‐1 monocytes	Direct 4 co‐cultures: SMCs seeded into culture wells and HUVECs & THP‐1on inserts. SMC + HUVEC SMC + HUVEC + AGEs SMC + HUVEC + THP‐1 SMC + HUVEC + THP‐1 + AGEs	48 h	Effect of glycol‐AGEs on SMC proliferation.	65
HCASMCs HCAECs THP‐1 monocytes	Direct 4 co‐culture models of the following: *Normal intima*: HCAECs on top of collagen type 1 matrix. *Atherogenesis*: Normal intima model + LDL in the culture medium. *Intimal xanthoma*: Atherogenesis model + THP‐1 monocytes on top of ECs. *Pathological intimal thickening*: Intimal xanthoma model + SMCs in the collagen matrix.	Up to 24 h	Effect of shear stress on a triple‐cell model of the atherosclerotic artery wall.	52

## 3 SPHEROID MODELS FOR ATHEROSCLEROSIS RESEARCH

A spheroid is a compact, solid mass of cells held together by interconnected proteins such as ECM‐located homophilic cadherins.^6^ Though both spheroids and organoids are 3D models, cell organization within spheroids depends on cell–cell aggregation and adhesion supported by cadherins, while cell organization in organoids depends on the self‐assembly of differentiated cells responding to physical and chemical factors introduced to make them resemble the native organ.^73^, ^74^ Spheroids are favored as *in vitro* atherosclerosis models because they support the study of cell–cell signaling and cell‐ECM interactions, and can be studied with established techniques to obtain qualitative and quantitative data.^28^

To design a robust spheroid model, both intrinsic and extrinsic factors should be considered. Intrinsic factors relate to inherent cell traits, such as their nature (primary or immortalized), their behavior in culture (adherent or suspended), morphology, proliferation rates, viability, and lifespan. Conversely, extrinsic factors refer to the cell culture parameters (such as culture duration and media choice) and choice of fabrication technique.^75^ Existing reviews of other scaffold‐based models for cardiovascular disease modeling^31^ and the common spheroid fabrication methods can be consulted for further insights.^27^, ^32^, ^33^, ^75^, ^76^ Due to their three‐dimensional nature, gradients which cannot be observed in monolayer cultures can exist in spheroids between the core and its periphery, affecting the transfer of oxygen, nutrients, metabolites, and intercellular signals.^32^ The gradients can also lead to heterogeneity in the spheroid construct, with cells being hypoxic or normoxic, quiescent or replicating, depending on their location.^16^, ^32^ Furthermore, as spheroid formation relies on cellular self‐aggregation which leads to compactness,^30^ they can be constructed with defined geometry through the optimization of the fabrication technique. Further optimization according to experimental needs may also be used to advance their scalability for high‐throughput screening.^32^

Spheroids offer a unique microarchitecture to study various aspects of cellular function under tunable conditions and a controlled environment.^77^, ^78^ Mesenchymal stem cell (MSC) spheroids have been used to improve the efficacy of MSC‐based therapeutics for tissue regeneration.^32^

Multicellular cardiac spheroids using primary cardiomyocytes (CMs), induced pluripotent stem cell‐derived cardiac fibroblasts (iCFs) and coronary artery endothelial cells (ECs) have also been developed as *in vitro* heart models.^86^ Similarly, multicellular cancer spheroid models have been used to study hypoxia, oxidative stress, cell invasion, migration, cell–cell crosstalk, and adapted for the high‐throughput screening of anticancer drugs.^87^ Certain physiological similarities exist between the tumor and atherosclerotic plaque microenvironments, such as endothelial dysfunction, upregulation of adhesion molecules, inflammation, and neovascularization.^4^ Furthermore, recent findings have reported similarities in etiology and molecular pathways between cancer and atherosclerotic disease.^88^, ^89^ Therefore, it is thought that studies investigating atherosclerotic disease progression could benefit from leveraging spheroid models, as the latter have been adopted successfully for *in vitro* cancer studies.^90^, ^91^, ^92^

To date, both monoculture and co‐culture spheroids have been developed to investigate various atherosclerotic phenomena such as intimal lesion formation and thickening, inflammation, lipid accumulation, and efferocytosis in atherosclerosis using macrophages, foam cells, and SMCs.^79^, ^80^, ^81^, ^82^, ^83^, ^84^, ^85^ However, compared to their 2D counterpart, there is a paucity of spheroid atherosclerosis models, attributed to the relative recency of spheroid model adoption for atherosclerosis phenomena modeling. A summary of the attributes of existing spheroid models is provided in Table 5 and key findings are further discussed in the following sections. Taken together, these studies show that spheroid models are promising models to replicate the 3D architecture of the plaque microenvironment, study the responses evoked by cellular interactions (such as morphological changes, gene, protein, or cytokine expression), and investigate the efficacy of novel therapeutic agents.

**TABLE 5:** Spheroid models utilized for atherosclerosis‐phenomena modeling.

Phenomena modeled	Study aim	Cells	Technique	Investigations	Ref.
Intimal thickening in atherosclerosis.	Imitate the arterial wall by the development of smooth muscle cell (SMC) spheroids.	Donor‐obtained arterial wall SMCs	Agarose‐coated microwells	Structural changes in spheroid morphology. Effect of serum on spheroid cultures.	79
Organotypic organization & differentiation of vascular SMCs in response to environmental or biochemical stimuli.	Compare the effect of the 3D microenvironment on the transcriptomic & proliferative ability of VSMC spheroids v/s 2D monolayers.	HUASMCs	Hanging droplet	Effect of the 3D environment on gene expression, SMC differentiation & proliferation.	80
Inflammation in atherosclerosis.	Examine the anti‐inflammatory effects of a simvastatin‐loaded nanoliposomal formulation using a foam cell spheroid model.	Foam cells (THP‐1 derived macrophages incubated with ox‐LDL).	Hanging droplet	Spheroid characterization (Oil Red O staining, cell viability). Cytokine expression post‐treatment of spheroids with nanoliposomes.	81
Inflammation & lipid accumulation in atherosclerosis.	Investigate the anti‐inflammatory potential of fluocinolone acetonide (FA) in atherosclerosis treatment.	Foam cells	Hanging droplet	Spheroid characterization (Oil Red O staining, cell viability) Cytokine expression post‐drug treatment (inflammatory & lipid‐accumulating cytokines).	82
Late‐stage atheroma development	Create a pseudo‐plaque (“ps‐plaque”) architecture to model the late stage fibroatheroma.	THP‐1 monocytes & macrophages Dendritic cells Human umbilical vein myofibroblasts.	Hanging droplet	Gene expression Cell viability & necrosis within the pseudo‐plaque. Comparative study between the pseudo‐plaque & human atherosclerotic plaques.	83
Efferocytosis in atherosclerosis.	Investigate the efferocytotic ability of lipid‐laden multicellular spheroids.	Macrophages SMCs ECs	Agarose micro‐mould	Spheroid morphology & cell distribution. Apoptotic staining of spheroids.	84
VSMC‐mediated neointima formation.	Utilize a machine‐learning based framework to identify morphological subpopulations of drug‐treated VSMC spheroids.	Primary human vascular SMCs Primary mouse SMCs	Hanging droplet	Spheroid formation and imaging over time. Morphology changes post treatment with inhibitors of FAK phosphorylation and Rac, Rho, & Cdc42 activation. Unsupervised spheroid morphology learning. Supervised (deep learning) of spheroid images.	85

### 3.1 Monoculture spheroid models of atherosclerosis

The earliest report on the development of spheroids for atherosclerosis studies dates back to 1994, when arterial smooth muscle cells were used to create spheroids and their structural organization was compared with that of the native arterial wall.^79^ These spheroids were fabricated using microwells coated with agarose, and cultivated in high serum, low serum, and serum‐deprived media. Transmission electron microscopy images revealed the formation of spheroids with an external shell‐like layer and an internal porous‐core, which disintegrated in the absence of fresh serum and media.^79^ This study served as the earliest report about the prospects of spheroids as *in vitro* models of atherogenesis and provided insights into the behavior of smooth muscle cells in a 3D environment.

**FIGURE 5:**
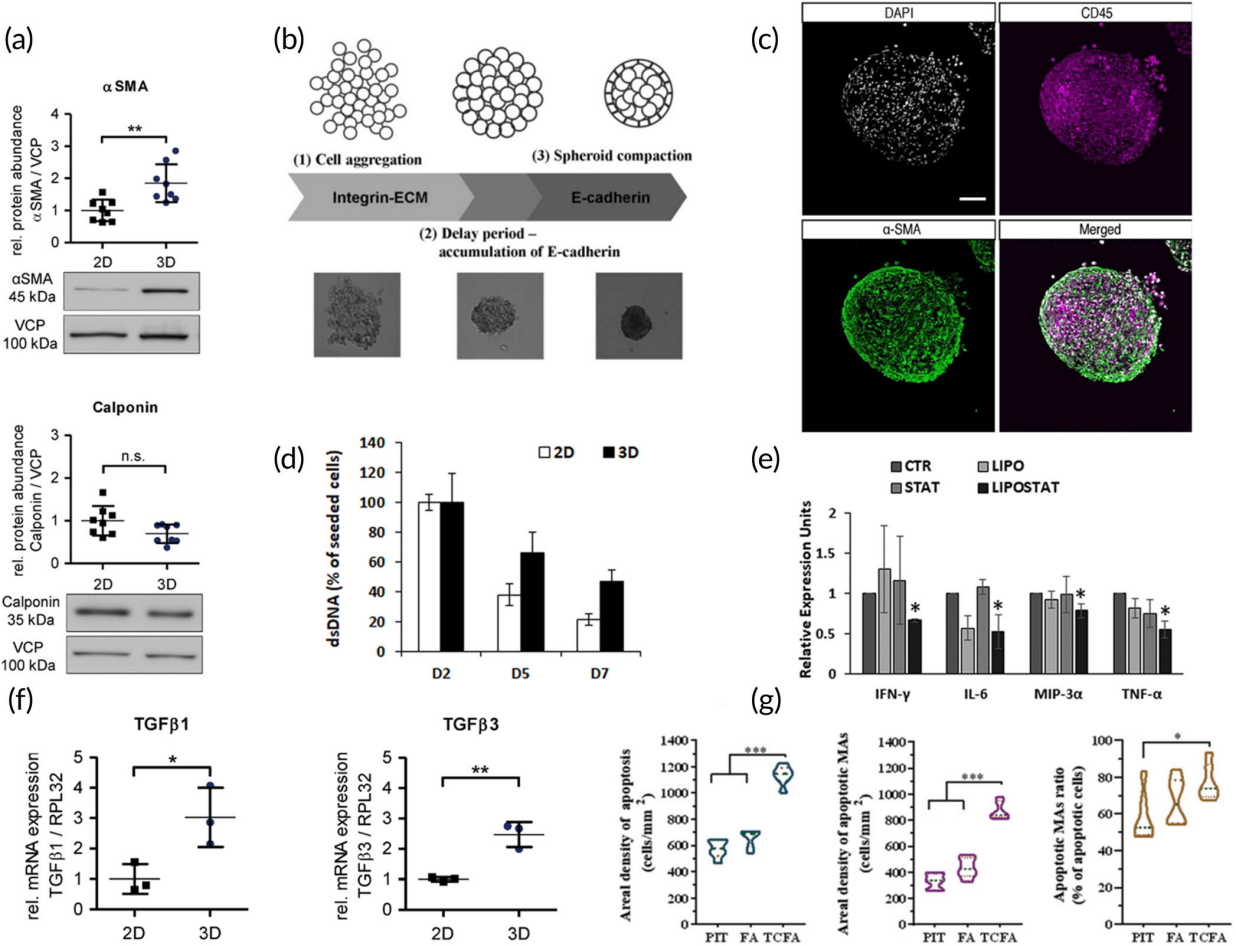
Spheroid models for atherosclerosis research. (a) Protein expression of α‐smooth muscle actin and calponin by 2D SMCs and SMC spheroids. (ns: not significant, **p < 0.01, *n* = 7–8. Reference protein for immunoblot analysis: Valosin containing protein (VCP). Reproduced from Reference 80. (b) A schematic representation of the spheroid formation process. Reproduced from Reference 75. (c) The inner architecture of a “pseudo‐plaque” showing the myofibroblasts at the periphery (α‐SMA stained in green) and myeloid cells at the core (CD45+ stained in magenta). Nuclei stained with DAPI. Scale bar: 100 μm. Reproduced from Reference 83. (d) Quantitative measurement of dsDNA via PicoGreen Assay to evaluate foam cell spheroid viability relative to 2D foam cells after 2, 5, and 7 days of culture. Reproduced from Reference 81. (e) Drug screening using foam cell spheroids. Reproduced from Reference 81. Spheroids were incubated with liposomes (LIPO‐180 μM), simvastatin free drug (STAT‐10 μg/ml), simvastatin‐loaded liposomes (LIPOSTAT‐10 μg/ml) or untreated (CTR), and the release of inflammatory cytokines was measured. IFN‐γ: Interferon‐gamma. IL‐6: Interleukin‐6. MIP‐3α: Macrophage inflammatory protein‐3‐alpha. TNF‐α: Tumor necrosis factor‐alpha. (f) mRNA analysis of TGFβ1 and TGFβ3 expression in 2D monolayer SMCs and SMC spheroids by qPCR (unpaired Student's *t*‐test, *p < 0.05, **p < 0.01, *n* = 3). Reproduced from Reference 80. (g). Increase in apoptotic activity in macrophage‐SMC‐EC spheroids corresponding to plaque stages. (*n* = 6 for each analysis; data analyzed by one‐way ANOVA. *p ≤0.05, ***p ≤0.001). Reproduced from Reference 84. FA, stable fibroatheroma; PIT, Pathological intimal thickening; TCFA, Thin cap fibroatheroma.

More recently, human umbilical artery smooth muscle cell spheroids were used to investigate the role of TGF‐β signaling on VSMC cell growth.^80^ Results revealed that spheroid formation was associated with the expression of native SMC markers (α‐SMC actin and calponin), and that VSMC proliferation was inhibited by the upregulation of TGF‐β1 and TGF‐β3 mRNA, similar to what one obtains in the intact native intima (Figure 5). Taking this further, SMC spheroid fabrication was complemented with a machine learning framework to elucidate morphological changes spheroids undergo post‐treatment with FAK, Rac, Rho and Cdc42 inhibitors.^85^ Images of the fabricated spheroids were successfully utilized for machine‐learning based image segmentation and subsequent data clustering analysis, to confirm the presence of heterogeneous subpopulation profiles. These studies show spheroids as biomimetic models which can be complemented with computational tools to obtain useful insights. Similarly, foam cell spheroids have been fabricated to screen anti‐inflammatory glucocorticoids (dexamethasone and fluocinolone acetonide)^82^ and simvastatin drug‐loaded liposomes.^81^

Common to both studies were the fabrication of the spheroids via the hanging droplet method and the use of foam cells obtained by the incubation of THP‐1 derived macrophages with ox‐LDL. Interestingly, the foam cell spheroids also expressed a higher viability compared to 2D foam cells after 7 days of culture, and served as a robust model to screen the efficacy of the simvastatin‐loaded liposomes, in comparison to free drug (Figure 5).^81^

### 3.2 Co‐culture spheroid atheroma models

The late stage of atheroma formation, that is, the formation of a “thin cap fibroatheroma (TCFA)” was modeled by Mallone et al., using spheroids made of myeloid cells and myofibroblasts (6:1 co‐culture ratio), known as “pseudo‐plaques.”^83^ The viSNE algorithm was then utilized to interpret the pseudo‐plaque flow cytometry data and classify various cell populations in the spheroids such as plasmacytoid and pre‐classical dendritic cells. The stratified structure of the spheroids revealed the myofibroblasts located at the periphery and the myeloid CD45+ cells at the core (Figure 5). This architecture is similar to that of the native fibroatheroma, which has macrophage‐derived foam cells and dendritic cells embedded in a collagenous matrix and an exterior thin layer of fibrotic cells. To model the various stages of plaque formation, from early pathological intimal thickening (PIT), stable fibroatheroma (FA) formation, to TCFA, spheroids were also fabricated by varying the concentrations of ox‐LDL and the macrophage: SMC co‐culture ratios (PIT‐3:1, FA‐6:1, TCFA‐9:1).^84^ Results showed that apoptosis in the spheroid cores corresponded to the stage of the plaque, with more apoptosis occurring in the TCFA model compared with the PIT model (Figure 5). Collectively, these studies provided insights into the prospects of multicellular spheroids to replicate early to late‐stage atherosclerotic disease phenomena.

### 3.3 3D Cell‐laden scaffold models

In a cell‐laden scaffold model, the cultured cells are retained in a fibrous or porous scaffold which serves as the supporting framework.^93^ Porous scaffolds created using hydrogels favor the encapsulation of RGD peptides and vascular endothelial growth factor (VEGF) to promote the differentiation of the retained cells.^94^ Upon review of the literature, it was observed that certain 3D cell‐based scaffold models have been developed for atherosclerosis modeling. These scaffold models have been developed to study phenomena which are relevant to atherosclerosis progression, such as endothelial dysfunction, angiogenesis, and neointima formation, as described in this section. ECM‐based hydrogels such as collagen 1 and matrigel have been reported for the fabrication of 3D angiogenesis models. Some examples include an anastomosing capillary model developed using capillary ECs cultured in a 3D collagen matrix *in vitro*,^95^ and a “radial invasion of matrix by aggregated cells” (RIMAC) model developed with bovine aortic ECs which migrated radially within collagen type 1 matrices.^96^ Similarly, the gelatin methacrylate (GelMA) hydrogel has been used to develop a 3D artery model, created with HUVECs and SMCs, to investigate the role of zinc ions in atherosclerosis progression,^97^ while a neointima model has been created with THP‐1 derived foam cells and collagen 1.^98^ In addition, the effects of ECM on immune cells and the *Aggregatibacter actinomycetemcomitans* bacteria (associated with periodontal disease) on endothelial dysfunction have been studied using collagen 1 scaffold models.^99^, ^100^ Summarily, existing reviews about the general prospects of scaffold models (particularly the use of decellularized scaffolds for the development of models with non‐immunogenic microenvironments) have been published and can be further consulted for additional insights.^101^, ^102^

## 4 DEVELOPMENT OF ROBUST CELL‐BASED MODELS: DESIGN PARAMETERS TO CONSIDER

Cell‐based models are favored for their ability to serve as platforms for high‐throughput screening in the development of new therapies and provide a valuable means for the multiparametric investigation of biological mechanisms in a reproducible and controllable manner.^24^ Important factors which influence the design of robust cell‐based models are further discussed.

### 4.1 Nature of the plaque to be modeled

Atherosclerotic plaques can be broadly divided into two groups: “vulnerable or unstable” and “stable” plaques. Vulnerable plaques are characterized by active inflammation, large and rich lipid cores and thin fibrous caps.^4^, ^103^ They are generally, prone to rupture, and are associated with adverse cardiovascular events. In contrast, stable plaques possess thicker fibrous caps and generally remain clinically silent until they cause severe luminal stenosis.^4^ A model of the vulnerable atherosclerotic plaque would be desirable to gain insights into the molecular mechanisms which predispose it to rupture, whereas a model of the stable plaque would be relevant to gain insights into the progression of stenosis. Understanding the features of the lesion to be mimicked will influence the choice of parameters such as cell types and extracellular matrix composition. The histopathologic features of atherosclerotic lesions, based on their level of progression, calcification, and fibrosis have been described in the literature and can be consulted to gain a better understanding of how these features influence the model to be developed.^104^, ^105^, ^106^

### 4.2 Pathological process to be investigated

**FIGURE 6:**
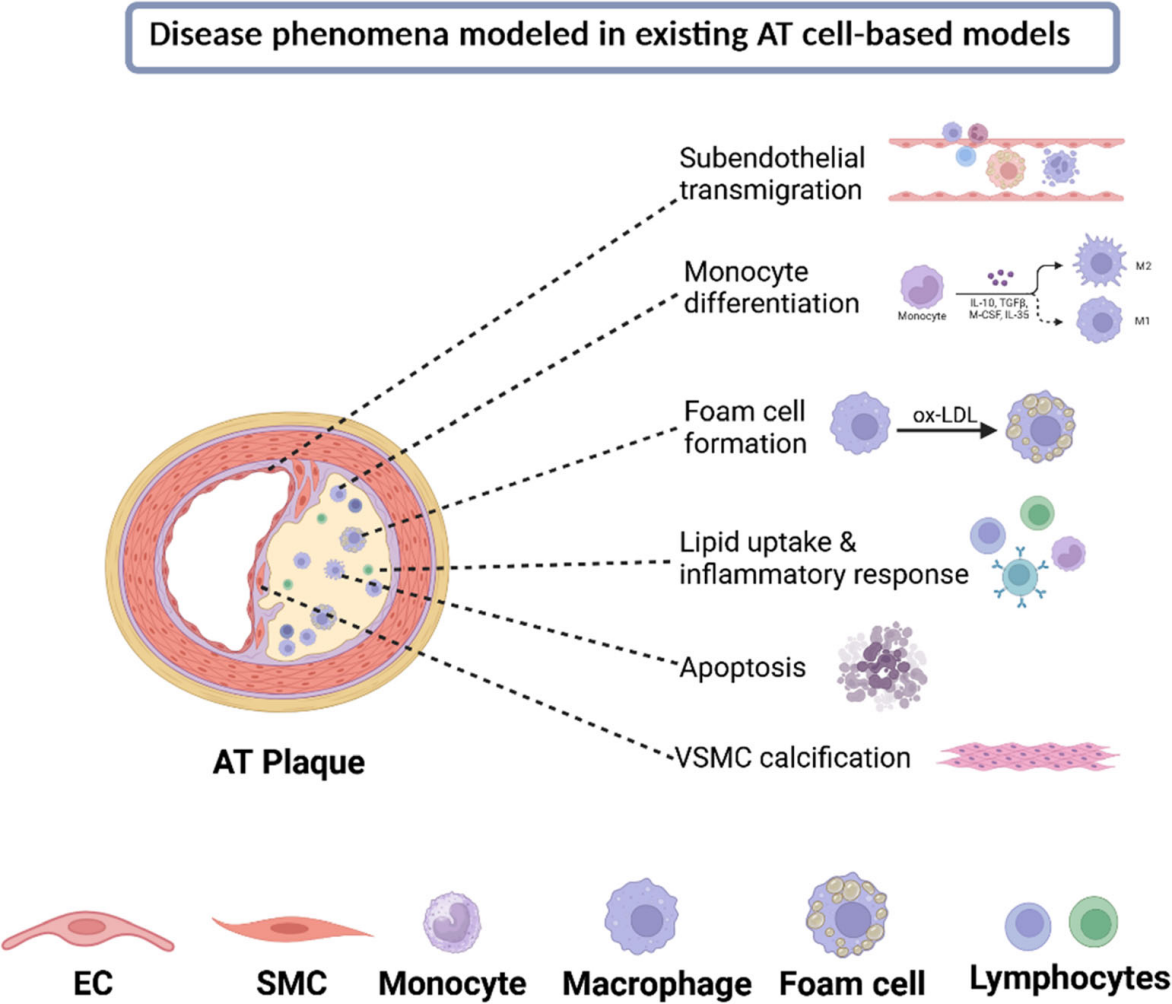
Atherosclerotic disease processes which have been studied using cell‐based models.

As a multifaceted disease, atherosclerosis involves multiple pathological processes, which can be comprehensively elucidated through the informed choice of a cell‐based model. These processes include endothelial activation, recruitment of immune cells to the site of endothelial injury, lipid accumulation by macrophages within the plaque, inflammation, SMC proliferation and migration, calcification, efferocytosis and apoptosis.^103^, ^107^ An overview of the disease phenomena which have been studied in existing cell‐based models is provided in Figure 6.

### 4.3 Choice of cell types

Cell types which have been used for the design of cell‐based models for atherosclerosis studies include monocytes, macrophages, foam cells, smooth muscle cells and myofibroblasts.^6^, ^36^ Other cells which have also gained attention for their role in modulating the microenvironment of atherosclerotic disease include dendritic cells^108^ and leukocytes (including neutrophils and lymphocytes).^109^ When the endothelium is dysfunctional, adhesion molecules and chemokines which attract and allow attachment of monocytes to the site of endothelial injury are increased.^31^ Endothelial cells (ECs) exposed to disturbed blood flow and low shear stress tend to exhibit a higher permeability, which promotes the recruitment of inflammatory cells into the sub‐endothelial space.^110^ Thus, existing cell‐based models of atherosclerosis have also investigated the interactions between ECs and inflammatory cells, as well as monocyte transmigration into the subendothelial space.^36^, ^37^, ^39^

Monocytes readily access the site of endothelial injury during the onset of atherosclerotic disease and have been represented in cell‐based models with immortalized THP‐1 cells. These suspension cells are derived from the peripheral blood of a patient with acute monocytic leukemia and are used to study immune and inflammatory responses in *in vitro* cell‐based models of atherosclerosis. Although being monocytes, THP‐1 cells can be differentiated by chemical agents such as phorbol 12‐myristate‐13‐acetate (PMA), 1α, 25‐dihydroxyvitamin D3 or macrophage colony stimulating factor (M‐CSF) to become macrophages.^111^ The advantages of the THP‐1 cell line over peripheral blood mononuclear cells include rapid growth, ease of use, robustness, as well as longevity and minimal heterogeneity.^111^ It has also been reported that THP‐1 cells line are similar to native human monocytes as regards morphology, the expression of secretory products, membrane antigens, oncogene expression, and lipid metabolism.^112^

Likewise, macrophages play an active role in the progression of atherosclerosis. Within the plaque microenvironment, macrophages are produced by the differentiation of monocytes which have been trapped beneath the endothelial space. Macrophage differentiation from monocytes may be prompted by macrophage colony‐stimulating factor (M‐CSF) or granulocyte‐macrophage colony‐stimulating factor (GM‐CSF).^35^ Due to their heterogeneity and plasticity, macrophages can be polarized to a broad array of phenotypes which are conventionally classified as pro‐inflammatory (M1) or anti‐inflammatory (M2).^35^ M1 macrophage polarization is typically induced using interferon‐γ (IFN‐γ) and lipopolysaccharides (LPS), whereas M2 macrophage polarization is typically induced with cytokines (interleukin 4 [IL‐4] and interleukin 13 [IL‐13] or interleukin 10 [IL‐10]).^35^ More information about the chemokines and cytokines released by activated macrophages and the roles they play in inducing inflammation within the atherosclerotic plaque has been extensively described in the literature.^35^, ^113^, ^114^, ^115^ Other macrophage subtypes which play a role in atherogenesis include Mme (metabolic activated) macrophages, Mox (Heme Oxygenaase‐1 enzyme producing) macrophages, and M4 macrophages (activated by the platelet chemokine CXCL‐4).^116^

In contrast, foam cells are formed when macrophages in the sub‐endothelial space accumulate excess cholesterol due to dysregulated lipid metabolism and clearance.^117^ When macrophages are unable to clear out residual cholesterol from their cytoplasm, they develop a foam‐like appearance, hence the name “foam cells.”^35^ In atherosclerosis, foam cells are associated with plaque instability and the persistence of chronic inflammation, as they represent a type of dysfunctional macrophage.^117^ Due to their active role in the progression of atherosclerosis, foam cells have emerged as the target of choice cells for the therapeutic disease management and model design. Moreso, SMCs that reside within the tunica media in a contractile state in healthy blood vessels acquire a dedifferentiated, proliferative phenotype in the atherosclerotic environment.^35^, ^70^, ^118^ They migrate into the intima, where they display increased expression of collagen, proteoglycans, elastin and matrix metalloproteinases (MMPs).^35^ Furthermore, they can also take up lipids and become foam cells themselves.^119^ Therefore, SMCs have been incorporated into cell‐based models of atherosclerosis to achieve a high degree of model representativeness and study their functionality, growth, calcification, and apoptosis.^55^, ^56^

### 4.4 Inclusion of relevant non‐cellular factors

Non‐cellular components also play a key role in plaque growth and can be incorporated in the design of biomimetic cell‐based models. Two noteworthy examples are oxidized low‐density lipoprotein (ox‐LDL) and the extracellular matrix (ECM). Both macrophages and SMCs uptake ox‐LDL through their scavenger receptors and contribute to the growth of the fatty core.^35^, ^117^ Conversely, the fibrous cap of the atherosclerotic plaque is stabilized by the extracellular matrix (ECM) produced by SMCs.^113^ This ECM consists of up to 60% collagen,^99^ proteoglycans, and elastins, and is degraded by matrix metalloproteinases (MMP‐1, MMP‐2, MMP‐3, and MMP‐9).^35^ Collagen‐based scaffolds can be used to study ECM mechanics (stiffness and rigidity) and adapted to recapitulate early‐ and late‐stage atheroma microenvironments.^99^ The influence of ECM stiffness on SMC phenotype (morphology, proliferation, and α‐actin expression) has been investigated using ECM scaffolds created with Type I collagen (0.25, 2 and 6 mg/ml), Type 1 collagen supplemented with laminin 0.5 mg/ml and Type 1 collagen supplemented with fibronectin 0.5 mg/ml.^120^ Likewise, other atherosclerotic phenomena such as ox‐LDL uptake by THP‐1 derived macrophages, cytokine and gene expression have been measured using collagen‐based scaffolds.^99^

## 5 PERSPECTIVES AND OUTLOOK

**FIGURE 7:**
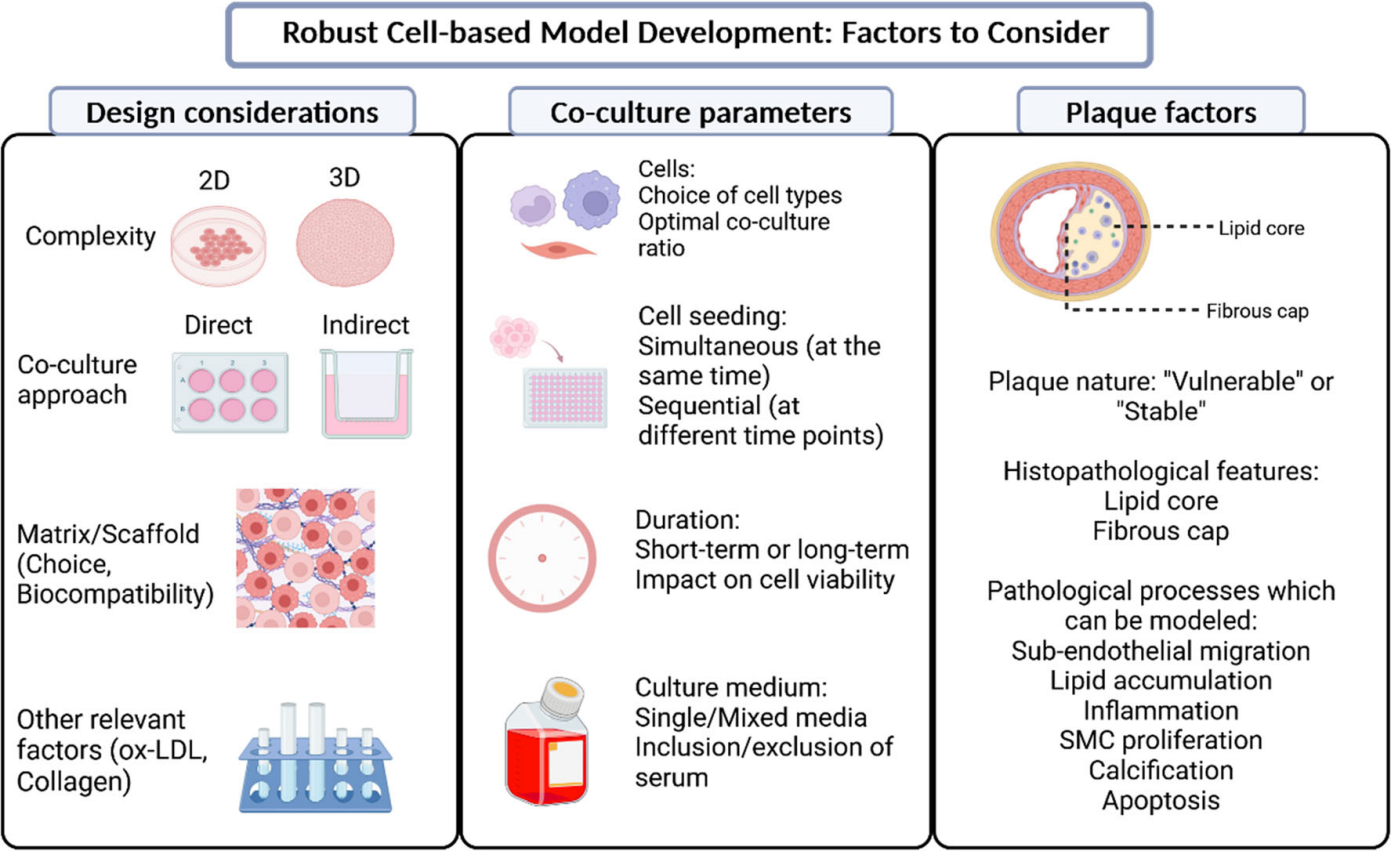
Multifaceted factors to consider for robust model design. Each model should be tailor‐designed with a careful understanding of the important components to incorporate, based on its intended aims.

To design a biomimetic, cell‐based model, understanding the plaque pathophysiology is crucial.^113^, ^121^ Knowledge about lipid accumulation within the plaque, changes in plaque architecture, the target cells which advance plaque‐based inflammation, and information about the genes and cytokines upregulated during plaque growth should be clearly defined and be included in the design process. As shown in this review, existing cell‐based models have most commonly adopted the use of monocytes, macrophages, SMCs and ECs exposed to the influence of ox‐LDL and/or ECM to obtain information about cell adhesion, migration, proliferation, and phenotypic expression. As crosstalk and interactions between these cells contribute significantly to plaque growth,^122^ they have been incorporated in cell‐based models to create a microenvironment suitable for the study of the specifically investigated atherosclerotic phenomena. Furthermore, it is important to clearly define the specific disease phenomena being investigated, as this will influence the choice of subsequent design parameters (such as cell culture approach [direct or indirect], the choice of cells to include in the model, the choice of non‐cellular factors known to promote atherosclerotic disease progression [such as ox‐LDL], and other co‐culture factors [co‐culture ratio, duration, media, and ECM]).^123^ Taken together, such a holistic approach will aid the development of cell‐based models which enhance our understanding of atherosclerotic disease (Figure 7). Based on the most recent literature and emerging new technologies and tools, these recommendations can be included in the design of atherosclerosis cell‐based model development to further advance knowledge in the field.

### 5.1 Expanding the scope of cell‐based models

In this review, we showed that cell‐based models play a significant role in study cellular and molecular mechanisms, model disease phenomena, screen molecular targets and investigate the efficacy and toxicity of lead molecules,^32^, ^33^, ^35^ and more specifically to model various stages of atherosclerotic disease progression. However, as the field emerges, future models should allow to investigate how age, cell mutations, sex, and genetics impact plaque development.^83^, ^124^

Investigators currently use different cell types (commercially obtained or donor‐sourced), co‐culture times, culture media, and co‐culture approaches to fabricate and maintain cell‐based models. Furthermore, batch‐to‐batch variations can occur during the preparation of hydrogel scaffolds for the fabrication of cell‐based scaffold models. These are barriers to achieving data reproducibility in the field, as variations to cell culture routines can impact cell quality and experimental outcomes.^125^ Thus, to enhance the reproducibility of results using cell‐based models, the standardization of cell culture and assay protocols should be advocated, as sophisticated methods for analysis, imaging and data quantification emerge.

Further research should also be devoted to study the impact of cell manipulation on cellular phenotypic and genotypic expression for model development, as marked changes will affect the model's ability to replicate the in vivo environment suitably. These improvements will help to consolidate cell‐based models as a reliable model to study atherosclerotic‐related phenomena and conduct the high‐throughput screening of novel drug molecules.^81^, ^82^

### 5.2 Investigating the intersection of atherosclerosis with other disease conditions

It is hardly ever the case that any human disease occurs in isolation, and atherosclerosis is no exception. Torres et al. have investigated the role of periodontal pathogenic bacteria on atherogenesis,^100^ and a review of *in vitro* models to study periodontopathogen‐induced atherogenesis has been conducted by Patra et al.^126^ Similarly, studies have linked gut microbiota with atherogenesis and atherosclerotic disease progression.^127^, ^128^, ^129^ In 2017, findings from the Canakinumab Anti‐inflammatory Thrombosis Outcome Study (CANTOS) trial, a randomized, double‐blind trial of canakinumab, a therapeutic monoclonal antibody targeting interleukin‐1β, showed that sole focus on lipidemia without managing the inflammatory response predisposed patients to a high risk of future adverse cardiovascular events.^43^ Other studies also confirm the key role of inflammation in atherosclerotic disease progression.^43^, ^113^, ^121^ As earlier highlighted in this review, cell‐based models have been developed to study how high glucose (HG) and advanced glycation end products (AGEs) can increase the production of pro‐inflammatory cytokines, as an example of investigating the impact of diabetic disease on atherosclerosis. Thus, future research into the development of novel cell‐based models can additionally examine how gut microbiota as well as the metabolic products, upregulated genes and proteins, and signaling pathways implicated in other disease conditions (such as diabetes and dyslipidemia), can contribute to exacerbating inflammation in atherosclerosis.

### 5.3 Developing more dynamic cell‐based models

**FIGURE 8:**
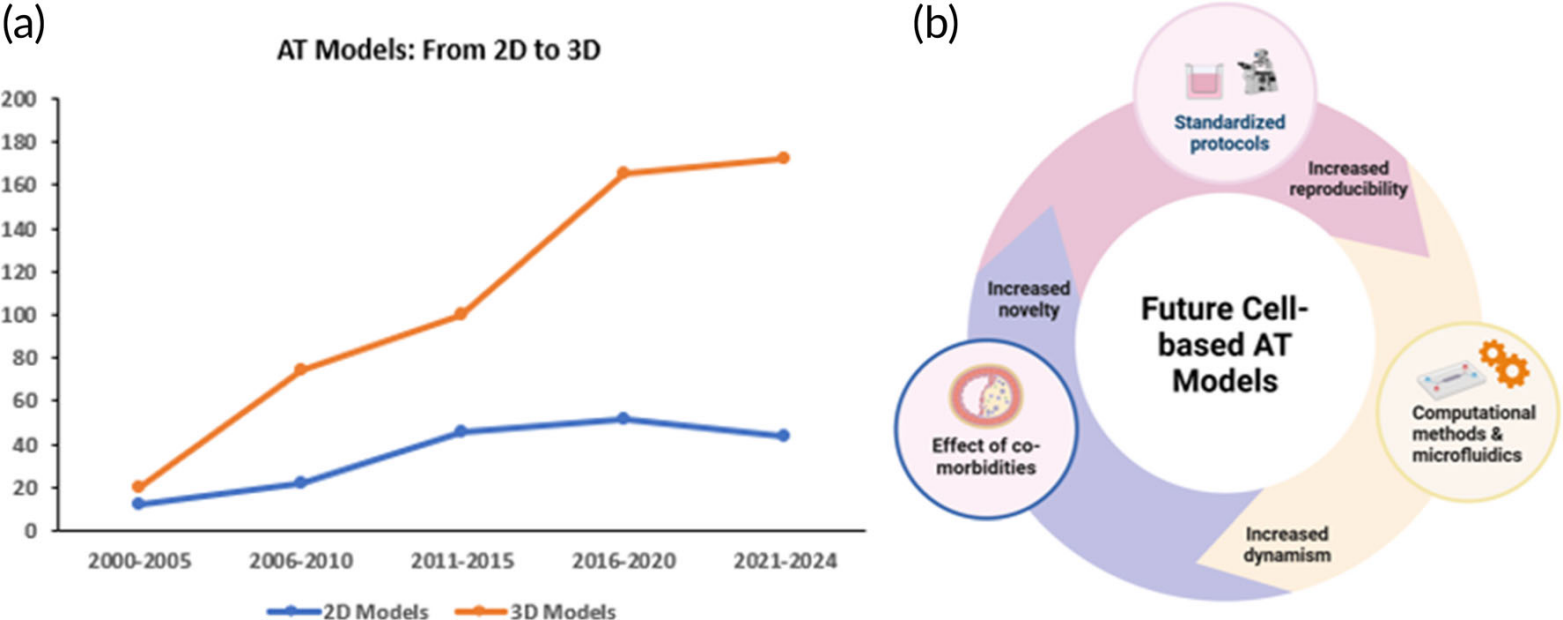
(a) The progressive shift from the development of 2D to 3D models of atherosclerosis. A search was performed on SCOPUS using the keywords “Atherosclerosis AND 2D models” and “Atherosclerosis AND 3D models.” Results were limited to publications written in the English language from 2000 to 2024. (b) Outlook on future research towards the development of robust cell‐based atherosclerosis models.

The shift towards the adoption of 3D models began within the last decade, and thus, there is enormous potential for advancement and further research (Figure 8). Several existing cell‐based models are static, so the possibility of multiplexing them with dynamic, fluidics‐based platforms (such as culture chamber models and microfluidic devices) to replicate the in vivo hemodynamic environment is one to explore.^124^ Compared to flow culture chamber models, microfluidic devices use smaller volumes, are more precise, and more versatile for vascular geometry design.^130^ As atherosclerotic plaques tend to develop in curved or branched arteries which are exposed to low shear stress or disturbed blood flow,^3^, ^124^ microfluidic devices can be used to replicate arterial architecture (curvature and stenosis), control fluid flow rate and shear stress on the endothelium with higher precision, or study the inflammatory responses of cells within microchannels.^131^

A microfluidic blood vessel model, fabricated to study how ECs and monocytes interact under stenotic conditions, has shown that adhesion molecules are more expressed in regions of high channel constriction.^132^ Conversely, a 3D microchannel model developed by Menon et al. with an EC‐lined lumen and a surrounding outer layer of aortic SMCs embedded in collagen was used to replicate the microvasculature of atheroprone vessels,^133^ while a co‐culture multichannel system containing human aortic SMCs and HUVECs was used to mimic the human artery and its inflammatory response when exposed to TNF‐α.^134^ A co‐culture model (containing human coronary artery ECs, SMCs, and THP‐1 monocytes) was exposed to shear stress and showed that inflammatory mediators (MCP‐1, IL‐1β, IL‐6, cathepsin L, and MMP‐1) were more expressed under low shear stress conditions (5 ± 3 dynes/cm^2^) compared with static conditions.^52^

Taking this further, future cell‐based model design can be complemented with computational methods to improve model dynamism. These could include mathematical modeling, computational simulation, or machine learning to study the effect of parameters such as flow rate, shear stress, curvature, and atheroprone stimuli on plaque formation.^85^, ^135^, ^136^, ^137^ Additional insights can be gleaned from studying the parameters which have been used to design other dynamic vascular models in existing literature,^123^, ^133^, ^138^, ^139^ to fabricate reproducible, and scalable cell‐based models. When taken together, these approaches will increase our understanding of plaque pathophysiology and increase our ability to design robust cell‐based models.

## 6 CONCLUSION

In this review, we discussed key parameters for the design of biomimetic 2D cell co‐culture‐based models and 3D spheroid models of atherosclerosis. These parameters include plaque pathophysiology, disease phenomena, choice of cell types and other relevant culture parameters, and a thorough understanding of them will aid the development of robust cell‐based models. As interest in the development of 3D atherosclerosis models only began within the last decade, there is currently a scarcity of spheroid models of atherosclerosis compared to their 2D counterparts. This is an avenue for promising future research, as novel co‐culture spheroid models can be developed to study how cell–cell interactions within the 3D microenvironment promote atherosclerotic disease. Furthermore, microfluidic devices can be developed to fabricate reproducible spheroid models in a dynamic environment and study cellular responses when the spheroids are exposed to shear stress and disturbed flow. Ultimately, it is anticipated that the perspectives shared in this review would promote the design of novel and reproducible atherosclerosis cell‐based models.

## AUTHOR CONTRIBUTIONS

**Ibukunoluwa Naiyeju:** Conceptualization; investigation; writing – original draft; writing – review and editing; visualization; methodology; formal analysis. **Stephanie Lehoux:** Conceptualization; methodology; writing – review and editing; formal analysis; supervision. **Maryam Tabrizian:** Conceptualization; funding acquisition; methodology; visualization; writing – review and editing; project administration; supervision; resources; investigation; formal analysis.

## CONFLICT OF INTEREST STATEMENT

The authors declare no conflict of interest.

### PEER REVIEW

The peer review history for this article is available at https://www.webofscience.com/api/gateway/wos/peer‐review/10.1002/btm2.10736.

## Data Availability

Data sharing not applicable to this article as no datasets were generated or analysed during the current study.
